# Translatability score revisited: differentiation for distinct disease areas

**DOI:** 10.1186/s12967-017-1329-y

**Published:** 2017-11-03

**Authors:** Alexandra Wendler, Martin Wehling

**Affiliations:** Institute of Experimental and Clinical Pharmacology and Toxicology, Clinical Pharmacology Mannheim, Faculty of Medicine Mannheim, Ruprecht-Karls-University of Heidelberg, Theodor-Kutzer-Ufer 1-3, 68167 Mannheim, Germany

**Keywords:** Translational science, Translatability scoring, Cardiovascular, Oncology, Psychiatric, Anti-infectives, Monogenetic orphans, Personalized medicine, Companion diagnostics, Animal models

## Abstract

**Background:**

Translational science supports successful transition of early biomedical research into human applications. In 2009 a translatability score to assess risk and identify strengths and weaknesses of a given project has been designed and successfully tested in case studies. The score elements, in particular the contributing weight factors, are heterogeneous for different disease areas; therefore, the score was individualized for six areas (cardiovascular, oncology, psychiatric, anti-viral, anti-bacterial/fungal and monogenetic diseases).

**Results:**

FDA reviews and related literature were used for modifications of the score with emphasis on biomarkers, personalized medicine and animal models. 113 new medical entities approved by FDA from 2012 through 2016 were evaluated and metrics obtained for companion diagnostics and animal models as starting points for author-based individualization of the score. Most drugs approved in this period were related to oncology (46%), while the approvals were lowest for psychiatrics (4%). The evaluation of the FDA package inserts revealed that companion diagnostics play an important role in every field except psychiatrics. Further the analysis of the FDA reviews showed the weakness of animal models in psychiatrics and anti-virals, while useful animal models were present for all other fields. Consequently the scoring system was adapted to the different fields, resulting in increased weights for animal models, biomarker and personalized medicine in oncology. For psychiatrics the weights for animal models, biomarker and personalized medicine were decreased, while the weight for model compounds, clinical trials and surrogate or endpoint strategy were increased. For anti-viral drugs weights for in vitro data and personalized medicine were increased, while the weight for animal models was decreased. Further, for anti-bacterial/fungal drugs weights for animal models and personalized medicine were increased. Weights were increased for genetics and personalized medicine and decreased for model compounds for monogenetic orphans.

**Conclusions:**

Adaptation of the score to different disease areas should help to support a structured and diverse approach to translation and encourage researchers in the private or public sectors to further customize the score.

**Electronic supplementary material:**

The online version of this article (10.1186/s12967-017-1329-y) contains supplementary material, which is available to authorized users.

## Background

Translational science is an important component of drug development aiming at the reduction of burgeoning timelines and costs mainly driven by late attrition in Phase II and III clinical trials [1]. It describes the transition of in vitro and in vivo data into human applications [2]. Understanding the biological evidence and clinical data supporting target selection is crucial and provides a basis for validation or invalidation of a scientific hypothesis [3]. One of several approaches to improve drug development is the casting of a translatability score published in 2009 to assess the availability and quality of in vitro and in vivo results, clinical data, biomarkers and personalized medicine issues [4]. Here we describe refined scoring templates tailored to reflect the diverging importance of scoring items in different therapeutic areas.

In brief, the original score [4] adds different items categorized between 1 and 5 and multiplied by a weight factor reflecting the importance of each item. The weight factors are generally higher for all items on human data, as positive clinical data are more indicative of successful drug development than in vitro or in in vivo animal data. Biomarkers play an important role in the score and a separate score for biomarkers is included. This biomarker score reflects animal and human data, their proximity to the disease, accessibility and test validity parameters such as sensitivity and specificity [5]. Any sum score of the translatability scoring system above four is indicative of fair to good translatability and low risk.

Other groups addressed the idea that a structured approach to guide and improve translation is much superior to the more inspirational ‘gut feeling’ approach of former times. Cook et al. [3] developed a system which basically includes the same criteria, the 5R framework. It was developed in reflection of AstraZeneca’s recent pipeline and seemed to tentatively improve success indicators of drug development after implementation.

An analysis of data from Phase II decisions for 44 programs at Pfizer revealed that success in Phase II clinical development is mainly related to drug exposure at the site of action, target binding and expression of functional pharmacological activity [6]. Their system called ‘three pillars of survival’ shares similarities with our score though being limited to a smaller number of items. The extent to which our system has influenced others in similar approaches is not clear; it has been cited in those papers above and earlier by a Pfizer group reporting on the highly structured and sophisticated data integrating approach described in [7]. It seems that it has at least supported the wider acceptance of structured and metric approaches in pharmaceutical decision making.

As prospective validation is hard to obtain (estimated study time > 8 years) and, thus, largely missing for all structured approaches so far, we retrospectively tested our score in eight case studies; they seemed to support the hypothesis that this score may indeed have prognostic power [8]. However, these cases from different disease areas already showed that the importance of in vitro and animal in vivo studies, clinical studies, biomarkers and personalized medicine may substantially vary between the disease areas. For example, considerable differences between oncologic and psychiatric diseases exist: as opposed to malignant diseases, high profile translational biomarkers are largely missing in psychiatric diseases. The underlying molecular mechanisms are increasingly well understood in oncology [9], which is not the case in psychiatric diseases. These deficiencies encumber drug development in latter area [10] resulting in a sparsely populated pipeline and low probability of approval (6.2%) [11]. In contrast, the molecular mechanisms of monogenetic orphan diseases are widely understood which is also reflected in the likelihood of approval (25.3% for all rare diseases, not only including monogenetics) [11]. Drugs against infectious diseases also have a high likelihood of approval (19.1%) [11] as their efficacy against microbes can be tested in vitro. Personalized medicine aspects are more important in the genetically heterogeneous fields of oncology compared to cardiovascular diseases for example.

## Methods

The translatability score published in 2009 [4] was customized to different areas of disease (cardiovascular, oncology, psychiatric, anti-viral, anti-bacterial/fungal and monogenetic orphans) on the basis of the FDA reviews, package inserts and related literature dealing with the drugs approved from 2012 to 2016.

For the evaluation of companion diagnostics (CDx) the FDA package inserts for the different drugs were evaluated. In our definition CDx are tests that provide information being essential for the safe and effective use of a corresponding drug or biological product in patients, including diagnosis, safety, efficacy and therapeutic monitoring. This does not apply to tests for basic safety monitoring like blood counts, liver enzymes etc. Tests in which specialized monitoring is necessary to safely apply a drug (such as coagulation factor determination for factor replacement therapy) were included in the assessment. Diagnostic tests were included if essential for the specific treatment (such as resistance testing for anti-viral drugs, the determination of receptor status in oncology and the detection of the corresponding genetic cause of a disease, especially in the monogenetic orphans).

For the evaluation of animal models the pharmacology reviews of the FDA were analyzed for cardiovascular, oncology, psychiatric and monogenetic orphans and the microbiology/virology. Only animal models for efficacy were analyzed and the number of animal models used was calculated. Further the percentages of total numbers were calculated for positive outcome prediction by animal models as stated in the FDA reviews and averages were displayed.

## Results

### Drug approvals in 2012–2016

FDA market approvals for the years 2012–2016 were analyzed to define the importance of the score items for the following six disease areas: cardiovascular, oncology, psychiatric, anti-viral, anti-bacterial/fungal and monogenetic orphan diseases (Additional file 1: Tables S1–S6).

**Fig. 1:**
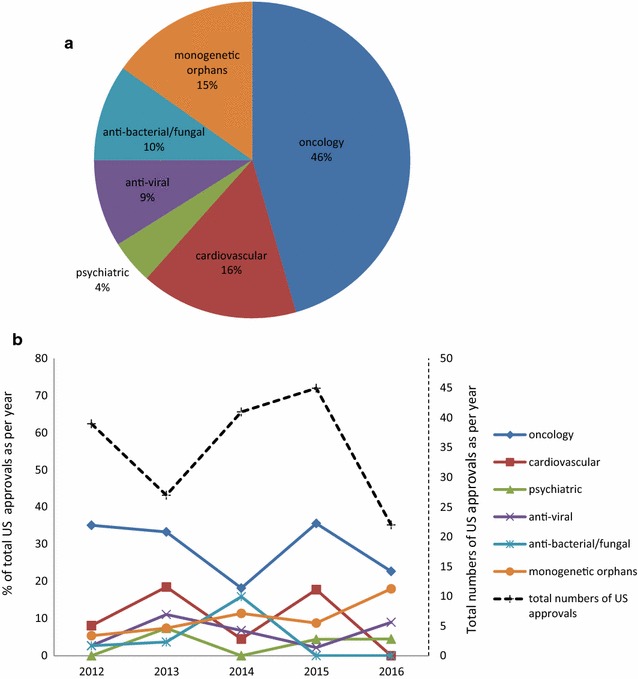
Drug approvals in 2012–2016. **a** Approvals in percent of total analyzed drugs as per therapeutic area. **b** US Drug approvals as per year. Primary y-axis (continuous lines): approval in percent of total US approvals as per year, secondary y-axis (broken line): total numbers of US approvals as per year

During that time span oncology approvals were most frequent (46%, Fig. 1a), while in psychiatrics only 4% of the total number were approved (Fig. 1a). This is in line with a relatively low likelihood of approval of 6.2% in psychiatrics determined for earlier years (2006–2015) [11]. Oncology drugs had a twofold higher rate of first cycle approval than psychiatric drugs, which had the lowest first cycle review approvals [11]. For infectious diseases the likelihood of approval is high with 19.1% [11], while numbers of approval in our sample (anti-viral: 9%, anti-bacterial/fungal: 10%) are lower than in cardiovascular (16%), and monogenetic orphan diseases (15%, Fig. 1a). The number of approved drugs depends on the number of drugs in the pipeline which is highest for oncology [12], therefore resulting in high approval numbers despite the low likelihood of approval (5.1% [11]). The astonishingly low likelihood of approval for oncology could be partially due to commercial decisions and portfolio prioritization [11, 13].

Oncology has the highest percentage of total US approvals in every analyzed year (Fig. 1b, primary y-axis, continuous lines). For the monogenetic orphans there is a trend towards more approvals (Fig. 1b, primary y-axis, continuous lines), which may be due to the intense support of the development of drugs against orphan diseases by the FDA.

Analyzing the different years there is a peak of total approvals in 2015, with a remarkable decrease in 2016 (Fig. 1b, secondary y-axis, broken line). In total (including all areas of disease) there have been only 22 approvals in 2016 compared to 30 in average in the years 2007–2015 [14]. The same trend was true for approvals by the EMA [15]. This may be due to the fact that some of the drugs which have been approved in 2015 have been originally scheduled for 2016, and for some, which have been scheduled to 2016 approval has been delayed, due to more complete response letters issued by the FDA. These letters were often addressing deficiencies in good manufacturing practice [16].

### Companion diagnostics are important in drug development

Biomarkers are important in many translational aspects and contribute 50% to the total translatability score if weight factors of related items (biomarker grading, biomarker development, biomarker strategy, surrogate or end point strategy) are added. Therefore, the use of biomarkers in the process of drug development has been evaluated in particular. Companion diagnostics play an increasingly important role in drug development and point to essential biomarkers as a *conditio sine qua non*, with a particular emphasis on personalized medicine.

Companion diagnostics is not a completely new phenomenon though very fashionable today. The use of tests to guide the effective and safe use of drugs is an essential part in the process of patient care and has been performed for a long time. Examples are glucose-6-phosphate-dehydrogenase deficiency testing and Rasburicase [15], or the identification of defective clotting factors for the diagnosis and treatment of patients with coagulation disorders. CDx was widely recognized only with the success of trastuzumab and imatinib [17].

There is no consistent definition of CDx [17]. For our definition see “Methods”. The definition of the FDA for CDx is stricter: A companion diagnostic is a medical device, often an in vitro device, which provides information that is essential for the safe and effective use of a corresponding drug or biological product, including identification of the right patient and monitoring response. The use of a companion diagnostic device with a particular therapeutic product is stipulated in the instructions for use in the labeling of both the diagnostic device and the corresponding therapeutic product [18].

**Fig. 2:**
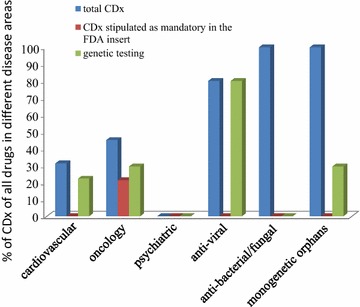
Analysis of FDA data on companion diagnostics (CDx) for drugs approved from 2012 to 2016. Total CDx: percentage of drugs with CDx as defined in text. CDx stipulated as mandatory in the FDA insert: percentage of drugs with CDx mandated by the FDA as stipulated in the package insert genetic testing: percentage of drugs with CDx representing genetic tests

In our extended definition, all assays mentioned in the FDA package insert dealing with efficacy, safety and/or monitoring were considered to be CDx, even if not explicitly mentioned and not found to be mandatorily tested by an FDA approved device. CDx have been found in all disease areas except psychiatrics (Fig. 2, column 1) as alluded to above. In our sample CDx stipulated mandatory in the FDA package insert were only present in oncology (Fig. 2, column 2). Our definition results in a high number of CDx for anti-infectives and monogenetic orphans, as susceptibility, hypersensitivity, resistance testing or underlying genetic cause should be tested for prior to therapy. The fraction of CDx is particular high for anti-viral drugs, as most of the approved drugs in the evaluated time period have been drugs against HIV or HCV (Additional file 1: Table S4), requiring resistance testing or genotyping, respectively. Therefore all tests for the anti-virals are genetic assays (Fig. 2, column 3). In contrast, there were no genetic tests for anti-bacterial/fungal drugs, as the recommended or necessary tests are dealing with susceptibility (antibiograms). For cardiovascular, oncology and monogenetic orphans, genetic tests are needed in some cases (Fig. 2, column 3) but also other tests, including enzymatic tests (Additional file 1: Tables S1, S2, S6), may be recommended. The high number of CDx in monogenetic orphans is due to the fact that the underlying genetic cause has to be analyzed using genetic or enzymatic tests to get a clear diagnosis.

In order to develop CDx, good understanding of the underlying mechanisms for the disease is important. If this is the case the likelihood for success is much higher so that it is not astonishing that the probability of successful transition from phase I to approval is 25.9% for drugs coming with a CDx test compared to 8.4% for drugs without predictive biomarker [11]. As evident in Fig. 2, the lack of CDx for psychiatrics is in line with the lack of biomarkers as a general feature of this disease area.

CDx may further facilitate the clinical studies as a smaller number of patients may be needed to show an effect, saving resources and time spent on clinical development [17]. The use of a biomarker in phase III increases the success to 62% compared to 28% overall success rate in phase III [11].

The potential of CDx in development will be mainly reflected in biomarker-related items of the scoring approach [5].

### Disease-area related differences in the predictivity of animal models

**Fig. 3:**
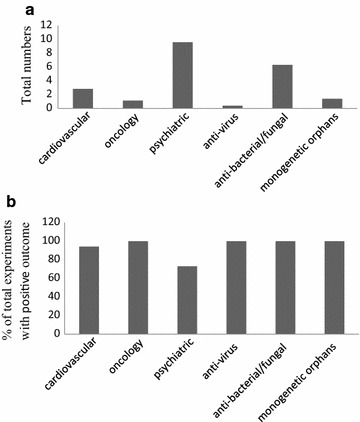
Animal models described in FDA reviews for drugs approved from 2012 to 2016. **a** Average total numbers of animal models used in different therapeutic areas as described in the FDA reviews. For xenograft studies experiments in same animals but using different cell lines were considered as one model. Different variants of nude mice were also considered as one model as the underlying principle is the same. Orthotopic or epitopic xenografts were considered as different models according to the classification of Ruggeri et al. [30]. **b** Percentages of total numbers were calculated for positive outcome prediction by animal models as stated in the FDA reviews; averages for disease areas are shown

Animal models are important to provide data on safety and efficacy (so-called animal-PoC, proof of concept) at the preclinical level; yet, there are large differences regarding availability and usefulness of animal models between disease areas. The average number of animal models reported for efficacy varied considerably between disease areas ranging from 0.4 to 8.4 (Fig. 3a). While relatively few different animal models could be found for oncology, cardiovascular, anti-viral and monogenetic orphan drugs, larger numbers of models have been reported for psychiatrics and anti-bacterial/fungal drugs. Despite the multitude of animal models used in psychiatrics, the average number of positive results translating into human findings was lowest in this area (Fig. 3b), indicating a low predictivity of animal models into clinical trials. In the other areas the contribution of animal models to translation was higher, as indicated by a greater rate of successful translation (Fig. 3b). The small number animal models tested for anti-viral drugs seems to reflect the fact that most of the approved drugs are treating HIV or HCV (Additional file 1: Table S4), which are highly specific for humans, and, therefore, animal models are not required by the FDA [19, 20]. The large number of animal models used to develop anti-bacterial/fungal drugs reflects the need for testing many different infection models including those on different sites of infection and sepsis (Additional file 1: Table S5).

Though not particularly prevalent in the sample of drug approvals analysed here, the weight factor for animal evidence should be increased (doubled or even tripled) case by case if primate studies were performed; they however cannot replace the value of human studies though their predictivity is certainly much higher than that of e.g. rodent studies.

### Adaptation of the translatability score to different disease areas

Based on the evaluation of the FDA reviews and related literature the translatability scoring system published 2009 [4] has been modified for different disease areas with particular emphasis on biomarkers and animal models as described above. Changes of weight factors were mainly driven by these aspects with weight changes for other aspects being adapted oppositely to keep the sum of weight factors at 100%. Changes in the weights differing by more than two points from the original are highlighted in italic (Table 1).

**Table 1:** Modified weight factors (in percent) for the items of the translatability score in the different disease areas

	Original = cardio-vascular	Oncology	Psychiatric	Anti-viral	Anti-bacterial/fungal	Monogenetic orphans
*Aspect*						
Starting evidence						
In vitro data including animal genetics	2	2	1	*9*	3	2
In vivo data including animal genetics	3	*1*	*1*	*1*	*5*	2
Animal disease models	3	*5*	*1*	*1*	*5*	2
Data from multiple species	3	*1*	*1*	*1*	*1*	2
Human evidence						
Genetics	5	5	4	4	4	*20*
Model compounds	13	*10*	*26*	12	12	*2*
Clinical trials	13	*11*	*26*	12	12	*10*
Biomarkers for efficacy and safety prediction						
Biomarker grading	24	*28*	*10*	23	*22*	23
Biomarker development	13	*17*	*5*	12	*11*	12
Proof-of-mechanism, proof-of-principle and proof of concept testing						
Biomarker strategy	5	5	*7*	5	5	5
Surrogate or endpoint strategy	8	*2*	*12*	7	7	7
Personalized medicine aspects						
Disease sub-classification and responder concentration	3	*7*	2	*9*	*9*	*9*
Pharmacogenetics	5	6	4	4	4	4
*Sum*	100	100	100	100	100	100

Deviations from the original = cardiovascular score by more than two points are in italic

Changes in the weights of the score have been performed according to the results of the FDA reviews and package inserts. Important weights were doubled (model compounds, clinical trials for psychiatrics), tripled (disease sub-classification and responder concentration, or quadrublicated (genetics for monogenetic orphans). Weights for points which are less important have been reduced. As the overall score needs to be 100 all other scores have been adapted accordingly.

The weights for cardiovascular diseases have been left unchanged, as the original score has been developed against a cardiovascular background. For oncology the weight of animal models has been increased, as our evaluation of the FDA reviews showed a good correlation between animal and clinical data (Fig. 3 and Additional file 1: Table S2). It is however of concern that a publication bias might exist for animal models as possibly not all negative results may have been discussed in the FDA documents. The weight for personalized medicine aspects and biomarkers have been increased in oncology in reflection of a large prevalence of CDx (45.1% of all oncology drugs, 23.5% stipulated as being mandatory by the FDA) this points to the high relevance of personalized medicine in oncology [21].

The weights for the starting evidence in psychiatric diseases have been reduced as reliable animal models and in vitro testing hardly exist (Fig. 3, Additional file 1: Table S3 and [22, 23]). The weights for model compounds which already made it into human application and clinical trials have been increased; this reflects the lack of animal models and suitable biomarkers rendering human evidence particularly important. Biomarkers for safety and efficacy are very difficult to develop in psychiatric diseases [10] and accordingly the weights of the related items (starting evidence, biomarkers for efficacy and safety prediction, biomarker strategy and personalized medicine) have been lowered. Surrogate or endpoint strategy scoring has been doubled, as the lack of animal models renders the surrogate or endpoint strategy especially important. The weights for personalized medicine aspects have been lowered since CDx are not found in this category (Fig. 2, Additional file 1: Table S3).

For virus-borne diseases the weight for in vitro data has been increased, while the weight of animal models has been decreased. Many viruses are highly specific for humans (for example HIV and HCV) and therefore reliable animal models are lacking. For example for HIV several animal models are available but none of them adequately reflects the human HIV [24]. This results in a greater importance of in vitro testing in this field. Further in vitro testing is well established for anti-viral (and anti-microbial) drugs [25]. Additionally the aspect of personalized medicine is quite important in this field e.g. HIV- or HCV-mediated diseases require special testing for resistance or genotyping, respectively (Additional file 1: Table S4).

For bacteria- and fungi-mediated diseases several reliable animal models exist (Fig. 3, Additional file 1: Table S5 and [26]), so that the weight for this aspect is higher than for anti-viral drugs. The aspect of personalized medicine is also quite important in this field due to susceptibility testing [27] and therefore the weight has been increased for this aspect.

For orphan genetic diseases animal models often exist (Fig. 3, Additional file 1: Table S6) as they do in cardiovascular diseases, so that the related weight has been left unchanged. The weight of human evidence, especially genetics, has been increased as by definition monogenetic orphan diseases always have a known genetic cause; this knowledge facilitates the translational process. The weight for model compounds has been lowered, since orphan drugs are mostly first in class drugs. Further the score for personalized medicine has been increased, as specific testing for the underlying genetic disease is performed in most cases.

## Discussion

The adaptation of the original translatability score reflects the varying requirements and challenges for the different disease areas in the translational process of drug development. In particular, it is obvious that biomarkers are indispensable for effective drug development, as they play a role in nearly all aspects of translation. Lack of knowledge on molecular mechanisms and the related absence of strong biomarkers result in low approval rates, as exemplified for psychiatric diseases.

The adaptations made should serve as suggestions on how the score might be modified before using it in a structured approach for a specialized area of biomedical research. The reasoning for these changes is based on an analysis of disease area-specific characteristics which may be done in an analogous way for other areas, and even adapted to the development of medical devices and diagnostics.

It, however, has to be emphasized that the changes of weight factors are subjective suggestions by the authors in reflection of the described analysis of drug approval data; no exact method could be identified to support these changes in a quantitative, metrical way. The approach chosen here, however, still seems to be useful to translate the area-specific peculiarities and encourage pharmaceutical scientists to modify them according to their own assessment and rating.

The rapidly emerging use of induced pluripotent stem cells may help to get valuable data in an early stage of drug development; these cells provide a rich source of patient-derived characteristics to screen for experimental drugs and profile them against patient-specific pathologies [28]. If proven to yield in highly translatable research results their contribution to the translatability score needs to be revised e.g. by increasing the weight factors relating to in vitro evidence.

It should also be mentioned that—beyond predictive approaches such as the one described here implying the usefulness of a general model—translation should also be driven from the statement and delineation of the particular problem to be solved. It should not solely based on paradigmatic solutions reflecting translational problems in other projects.

Predicting clinical responses to novel checkpoint inhibitors, cancer vaccines, or T cell redirection strategies is much more difficult due to the complexity of the substrate than predicting dependency on individual signal transduction pathways affected by well-characterized driver mutations [29]. The translational process never will be covered by a single static model or score, but must always be analyzed individually.

The scoring system should be applied after every major step of translation and adapted to the different requirements which may evolve in the different phases of preclinical and clinical development as well. Its application will not only lead to a numeric score for a project at a given stage, but also identify strengths (high partial scores) and weaknesses (low partial scores) of this particular project. Beyond its value to assess the risk of a portfolio of projects, it should guide further research and efforts to specifically develop and address the weaker aspects of a project. Thus, its prospective use will not only support portfolio decisions (kill or thrive) but also induce improvement strategies of high risk projects which should not be killed as they often promise the largest gains or leap innovations.

## Conclusions

- Drug development is highly complex and between disease areas or even within the same disease area important differences may exist; thus, the translatability score requires adjustments according to specific disease areas as provided here.
- A scoring system should help scientists to adequately address the cornerstones of translation, to intensify research in weak areas and ultimately develop a risk-balanced portfolio. As several similar approaches have been developed it is fair to assume that a structured approach to translation has been widely accepted to reflect the state-of-the art at least in industry.
- A prospective study on the impact of such a structured approach would be highly desirable but difficult to perform as a control group without structured approach would be hard to find at present.

## Additional file

**Additional file 1.** The tables in this file present detailed information on the CDx and animal models evaluated using the FDA documents for the different drugs. **Table S1.** Approvals for cardiovascular drugs 2012–2016. **Table S2.** Approvals in oncology 2012–2016. **Table S3.** Approvals for psychiatric drugs 2012–2016. **Table S4.** Approvals for anti-viral drugs 2012–2016. **Table S5.** Approvals for anti-bacterial/fungal diseases 2012–2016. **Table S6.** Approvals for monogenetic orphans 2012–2016.

## Abbreviations

FDA: US Food and Drug Administration
CDx: companion diagnostics
EMA: European Medicine Agency
HIV: human immunodeficiency virus
HCV: hepatitis C virus
