# Supplementary material for: Translatability score revisited: differentiation for distinct disease areas
Source: J Transl Med. 2017 Nov 3;15:226. doi: 10.1186/s12967-017-1329-y (PMC5670516; doi:10.1186/s12967-017-1329-y)
Supplement: Supplementary file 1 — Additional file 1. The tables in this file present detailed information on the CDx and animal models evaluated using the FDA documents for the different drugs. Table S1. Approvals for cardiovascular drugs 2012–2016. Table S2. Approvals in oncology 2012–2016. Table S3. Approvals for psychiatric drugs 2012–2016. Table S4. Approvals for anti-viral drugs 2012–2016. Table S5. Approvals for anti-bacterial/fungal diseases 2012–2016. Table S6. Approvals for monogenetic orphans 2012–2016. [file 12967_2017_1329_MOESM1_ESM.docx]

Additional file 1

Table S1: Approvals for cardiovascular drugs 2012-2016

| Drug | Description | Companion diagnostic | | Animal models for efficacy |
| --- | --- | --- | --- | --- |
| 2012 | | | | |
| apixaban | factor Xa inhibitor anticoagulant indicated to reduce the risk of stroke and systemic embolism in patients with nonvalvular atrial fibrillation, and for the prophylaxis of deep vein thrombosis in patients who have undergone hip or knee replacement surgery | no | | Effects of apixaban were evaluated in anesthetized rats, rabbits, and dogs. The models  included arterial-venous shunt thrombosis, tissue factor-stasis venous thrombosis,  FeCl_2_-induced vena cava thrombosis, FeCl_2_-induced carotid artery thrombosis,  electrically induced carotid arterial thrombosis and deep vein thrombosis.  Hemostasis was assessed in models of cuticle bleeding time, renal cortex bleeding time, and mesenteric bleeding time. (ten models)  (Pharmacology Review, Application Nr: 202155Orig1s000, FDA) |
| lomitapide | microsomal triglyceride transfer protein inhibitor indicated for the treatment of patients with homozygous familial hypercholesterolemia | Yes, homozygous familial hyper-cholesterolemia can be diagnosed by testing LDLc levels, a definitive diagnosis can be made by gene or receptor analysis. | | The cholesterol lowering activity of lomitapide was shown in Sprague-Dawley rats, Golden Syrian hamsters, and cynomolgous monkeys. (3 models) (Pharmacology Reviews, Application number:203858Orig1s000, FDA) |
| icosapent ethyl | ethyl ester of eicosapentaenoic acid (EPA) indicated as an adjunct to diet to reduce triglyceride (TG) levels in adult patients with severe hypertriglyceridemia | no | | In rats fed with high cholesterol diet ethyl EPA a significant reduction of cholesterol and TG levels was detected. (one model) (Pharmacology Reviews, Application Nr: 202057Orig1s000, FDA) |
| 2013 | | | | |
| riociguat | oral soluble guanylate cyclase stimulator for the treatment of pulmonary hypertension | no | | Normotensive rats, spontaneously hypertensive rats, and normal dogs were used to demonstrate riociguats hemodynamic effects of decreased mean arterial pressure, increased coronary blood flow and oxygen saturation. Also three models of induced pulmonary hypertension were used (mice, rabbits, rats). (six models) (Pharmacology Reviews, Application Nr: 204819Orig1s000, FDA) |
| mipomersen sodium | oligonucleotide inhibitor of apolipoprotein B-100 synthesis indicated for the treatment of patients with homozygous familial hypercholesterolemia | Yes, homozygous familial hyper-cholesterolemia can be diagnosed by testing LDLc levels, a definitive diagnosis can be made by gene or receptor analysis. | | No animal models were reviewed. (Pharmacology Reviews, Application Nr. 203568Orig1s000, FDA) |
| nimodipine | calcium channel blocker indicated to improve neurological outcome following subarachnoid hemorrhage | no | | No animal models were reviewed. (Pharmacology Reviews, Application Nr. 203340Orig1s000, FDA) |
| macitentan | dual endothelin receptor antagonist for the treatment of patients with pulmonary arterial hypertension | no | | Pharmacodynamic effects of macitentan have been investigated in normal rats and in rat disease models in which endothelin receptor plays a pathological role. (three models) (Pharmacology Reviews Application Nr:  204410Orig1s000, FDA) |
| polidocanol injectable foam | sclerosing agent indicated for the treatment of varicose veins | no | | New Zealand rabbits were used to model the situation where the vein is occluded, and to investigate the effect of polidocanol on vein endothelium. (four models, thereof one negative) (Pharmacology Reviews, application Nr: 205098Orig1s00, FDA) |
| 2014 | | | | |
| omega-3-carboxyclic acids | fish oil-derived mixture of free fatty acids for the treatment of patients with severe hypertriglyceridemia | no | | The lipid lowering effects of EPA have been observed in rodent, rabbits and primates.  (3 models) (Pharmacology Reviews, Application Nr: 205060Orig1s000, FDA) |
| vorapaxar | protease-activated receptor-1 antagonist for the prevention of cardiovascular events in high risk patients | no | | No animal models reviewed.  Only non-human primates or humans can be used to demonstrate  efficacy and anti-thrombotic activity of an antagonist  specific to human  PAR-1, as they express PAR-1 and PAR-4 on their platelets. (Pharmacology Reviews, Application Nr: 204886Orig1s000, FDA) |
| 2015 | | | | |
| ivabradine | hyperpolarization-activated cyclic nucleotide-gated channel blocker indicated for the treatment of chronic heart failure | | no | Bradycardic effect was shown in conscious normotensive rat  after single, intravenous or oral administration. Cardiac and regional haemodynamic effects were evaluated in  conscious Long Evans rats.  Hemodynamic and electrocardiographic effects were evaluated in anesthetized pigs. Improvement of angiogenesis and vascular protection by ivabradine was shown in a rat model of left ventricular dysfunction and chronic heart  failure.  The effect of chronic administration of ivabradine on left  ventricular contractile properties in a rat model of chronic heart failure was evaluated.  The effect of long-term therapy with ivabradine on LV  function and remodelling in dogs with chronic heart failure was evaluated.  The effect of chronic administration on survival and on the  development of heart failure in 132-AR transgenic mice was evaluated.  Further the effects of subchronic administration of ivabradine on  pressure overload-induced heart failure in rats was examined. (eight models, thereof two negative) (Pharmacology Reviews, Application number: 206143Orig1s000, FDA) |
| sacubitril and valsartan | neprilysin inhibitor and angiotensin II receptor blocker combination indicated to reduce the risk of death and hospitalization in patients with chronic heart failure | | no | Effects on the cardiovascular system have been evaluated in three different rodent disease models. (three models) (Pharmacology Reviews, Application Number: 207620Orig1s000, FDA) |
| sebelipase alfa | enzyme replacement therapy for the treatment of lysosomal acid lipase (LAL) deficiency | | Yes, test for biallelic pathogenic variants in LIPA or deficient LAL enzyme activity in peripheral blood leukocytes, fibroblasts or dried blood spots needed. | Administration resulted in the restoration of LAL enzymatic activity and reversal of the associated pathophysiology of LAL-/- rats. (one model) (Pharmacology Reviews, Application Nr: 125561Orig1s000, FDA) |
| cangrelor | intravenous P2Y_12_ platelet inhibitor indicated for use in patients undergoing percutaneous coronary intervention to reduce the risk of periprocedural thrombotic events | | no | In anaesthetized mongrel dog model of stenosed femoral artery induced by clamping for five minutes, cangrelor exhibited dose-related inhibition of thrombosis as measured by  inhibition of cyclic blood flow, with increase in prolongation of bleeding time.  In conscious beagle dogs infused continuously with cangrelor for 7 days, platelet aggregation in response to ADP was abolished.  In the dog model of occlusive thrombus and stenosis in the left circumflex coronary artery induced by electrical stimulation cangrelor reduced re-occlusion and cyclic blood flow in the coronary artery and reduced myocardial infarct size. (three models) (Pharmacology Reviews, Application Nr. 204958Orig1s000, FDA) |
| alirocumab | proprotein convertase subtilisin/kexin type 9 inhibitor monoclonal antibody for the treatment of heterozygous familial hypercholesterolemia, or patients with atherosclerotic heart disease who require additional lowering of LDL-cholesterol | | Yes, familial hyper-cholesterolemia can be diagnosed by testing LDLc levels, a definitive diagnosis can be made by gene or receptor analysis. | No animal models reviewed. (Pharmacology Reviews, Application Nr. 125559Orig1s000, FDA) |
| evolocumab | monoclonal antibody targeting proprotein convertase subtilisin/kexin type 9 (PCSK9) indicated for the treatment of patients with heterozygous familial hypercholesterolemia, homozygous familial hypercholesterolemia , or patients with atherosclerotic heart disease who require additional lowering of LDL-cholesterol | | Yes, homozygous familial hyper-cholesterolemia can be diagnosed by testing LDLc levels, a definitive diagnosis can be made by gene or receptor analysis. | *In vivo* administration of evolocumab in hamsters resulted in increased hepatic  LDLR protein, decreased serum non-HDL-C, and  total cholesterol. *In vivo* administration of evolocumab in male cynomolgous monkeys resulted in  decreased total cholesterol and serum LDL-C.  (two models)  (Pharmacology Reviews, Application Nr. 125522Orig1s000, FDA) |
| edoxaban | oral, once-daily factor Xa inhibitor anticoagulant indicated for the prevention of stroke and systemic embolism in patients with nonvalvular atrial fibrillation, and for the treatment of deep vein thrombosis, and pulmonary embolism | | no | An anti-thrombotic effect was shown in a rat arteriovenous shunt model, in a rat venous stasis model, a rat venous  thrombosis model, and a rat tissue factor  induced disseminated intravascular coagulation model. (four models) (Pharmacology Reviews, Application Nr. 206316Orig1Orig2s000, FDA) |
| selexipag | oral prostacyclin receptor agonist indicated for the treatment of pulmonary arterial hypertension | | no | No animal models reviewed. (Pharmacology Reviews, Application Nr. 207947Orig1s000, FDA) |
| 2016 | | | | |

Table S2: Approvals in oncology 2012-2016

| Drug | Description | Diagnostic companion | Animal models for efficacy |
| --- | --- | --- | --- |
| 2012 | | | |
| cabozantinib | kinase inhibitor that blocks abnormal kinase proteins involved in the development and growth of medullary cancer cells for treatment of metastatic medullary thyroid cancer | no | Cabozantinib inhibits tumor growth of various tumors in xenograft mice models.  (one model)  (Pharmacology Reviews, Application Nr. 203756Orig1s000, FDA) |
| vismodegib | for the treatment of basal cell carcinoma, works by inhibiting the Hedgehog pathway, a pathway that is active in most basal cell cancers and only a few normal tissues, such as hair follicles | no | Vismodegib was shown to be effective in the human colorectal carcinoma xenograft model.  (one model)  (Pharmacology Reviews, Application Nr. 203388Orig1s000, FDA) |
| axitinib | for the treatment of patients with advanced kidney cancer (renal cell carcinoma) who have not responded to another drug for this type of cancer | no | Axitinib was evaluated for its *in vivo* anti-tumor efficacy in a variety of tumor models  in rodent animals, including: 1) subcutaneously implanted human tumor xenograft  models; 2) orthotopically implanted and spontaneously metastatic human tumor  xenograft models; and 3) murine syngeneic tumor models.  (three models)  (Pharmacology Reviews, Application Nr. 202324Orig1s000, FDA) |
| pertuzumab | humanized monoclonal antibody indicated for the combination treatment of HER2-positive metastatic breast cancer, and for the neoadjuvant treatment of breast cancer | Yes, HER2  VENTANA HER2/neu (4B5) Rabbit  Monoclonal Primary Antibody assay needs to be applied as a companion diagnostic for detecting HER2 protein expression. | *In vivo* activity of pertuzumab was detected in patient-derived mammary, ovarian, and non-small cell lung cancer models.  (one model) (Pharmacology Reviews, Application Nr. 125409Orig1s000, FDA) |
| ingenol mebutate | first and only topical actinic keratosis therapy with 2 or 3 consecutive days of once-daily dosing | no | In mice models ingenol mebutate showed antitumoral activity. (one model) (Pharmacology Reviews, Application Nr. 202833Orig1s000, FDA) |
| regorafenib | oral multi-kinase inhibitor for the treatment of patients with metastatic colorectal cancer | no | Inhibition of tumor growth and angiogenesis was demonstrated in tumor xenograft models. (one model) (Pharmacology Reviews, Application Nr. 203085Orig1s000, FDA) |
| enzalutamide | androgen receptor inhibitor indicated for the treatment of patients with metastatic castration-resistant prostate cancer who have previously received docetaxel | no | Antitumor activity has been shown in mouse models of castrate-resistant prostate cancer. (one model)  (Pharmacology Reviews, Application Nr. 203415Orig1s000, FDA) |
| ziv-aflibercept | angiogenesis inhibitor for the combination treatment of patients with metastatic colorectal cancer | no | Ziv-aflibercept significantly inhibits tumor growth and angiogenesis, reduces tumor vessel density, and inhibits metastases in xenografts of various tumor types. (two models) (Pharmacology Reviews, Application Nr. 12541Orig1s000, FDA) |
| bosutinib | tyrosine kinase inhibitor for the treatment of Philadelphia chromosome positive chronic myelogenous leukemia | Yes, drug is indicated for Philadelphia chromosome positive patients tested by a BCR–ABL fusion test. | Xenograft models have been used successfully. (one model) (Pharmacology Reviews, Application Nr. 203341Orig1s000, FDA) |
| ponatinib | to treat adults with chronic myeloid leukemia and Philadelphia chromosome positive acute lymphoblastic leukemia | Yes, test of Philadelphia chromosome status. | SCID mice harboring subcutaneous tumor xenografts of the K562 human CML cell line expressing  native BCR-ABL, and a mouse model of CML containing the BCR-ABL T315I mutant which was injected intravenously to female SCID mice, were used to show antitumor activity. (two models) (Pharmacology Reviews, Application Nr. 203469Orig1s000, FDA) |
| carfilzomib | proteasome inhibitor indicated for the treatment of patients with multiple myeloma | no | *In vivo* efficacy was shown in HT-29 human colorectal adenocarcinoma  xenografts. (one model) (Pharmacology Reviews, Application Nr. 202714Orig1s000, FDA) |
| vincristine sulfate LIPOSOME injection | vinca alkaloid indicated for the treatment of adult patients with Philadelphia chromosome negative acute lymphoblastic leukemia | Yes, test of Philadelphia chromosome status. | Data not available. (Pharmacology Reviews, Application Nr. 202497Orig1s000, FDA) |
| omacetaxine mepesuccinate | first-in-class small molecule drug for the treatment of adult patients with chronic or accelerated phase chronic myeloid leukemia | no | Activity of omacetaxine mepesuccinate was investigated in a mouse model of chronic myeloid leukemia. (one model)  (Pharmacology Reviews, Application Nr. 203585Orig1s000, FDA) |
| 2013 | | | |

| afatinib | kinase inhibitor indicated for the treatment of patients with metastatic non-small cell lung cancer | Yes, approved concurrently with the therascreen EGFR RGQ PCR Kit, a companion diagnostic that helps to determine if a patient’s lung cancer cells express the EGFR mutations. | Treatment with afatinib reduced tumour volume in xenograft models based on a variety of cell lines. (one model) (Pharmacology Reviews, Application Nr. 201292Orig1s000, FDA) |
| --- | --- | --- | --- |
| ado-trastuzumab emtansine | HER2-targeted antibody and microtubule inhibitor conjugate indicated for the treatment of patients with HER2-positive, metastatic breast cancer | Yes, HER2-status must be tested by a FDA approved test. | No animal models reviewed. (Pharmacology Reviews, Application Nr. 125427Orig1s000, FDA) |
| trametinib | kinase inhibitor indicated for the treatment of patients with unresectable or metastatic melanoma with BRAF V600E or V600K mutations as detected by an FDA-approved test | Yes, BRAF V600E or V600K mutations must be detected by an FDA-approved test, for example companion diagnostic assay from bioMérieux S.A., THxID™-BRAF. | Efficacy was tested in various mouse  xenograft models of human cancer. (one model) (Pharmacology Reviews, Application Nr. 204114Orig1s000, FDA) |

| dabrafenib | kinase inhibitor indicated for the treatment of patients with unresectable or metastatic melanoma with BRAF V600E mutation as detected by an FDA-approved test | Yes, BRAF V600E mutation must be detected by an FDA-approved test, for example companion diagnostic assay from bioMérieux S.A., THxID™-BRAF. | Efficacy of dabrafenib was shown in CD-1 nu/nu mice bearing A375P  F11s tumor xenografts. (one model) (Pharmacology Reviews, Application Nr. 202806Orig1s000, FDA) |
| --- | --- | --- | --- |

| radium Ra 223 dichloride | alpha particle-emitting radioactive therapeutic agent indicated for the treatment of patients with castration-resistant prostate cancer | no | Effect of radium-223 dichloride on breast cancer bone metastases was successfully  studied in female athymic nude mice. (one model) (Pharmacology Reviews, Application Nr. 203971Orig1s000, FDA) |
| --- | --- | --- | --- |

| obinutuzumab | CD20-directed cytolytic antibody indicated for the combination treatment of patients with previously untreated chronic lymphocytic leukemia and follicular lymphoma | no | No animal models were reviewed. (Pharmacology Reviews, Application Nr. 125486Orig1s000, FDA) |
| --- | --- | --- | --- |
| ibrutinib | oral Bruton's tyrosine kinase inhibitor for the treatment of mantle cell lymphoma, chronic lymphocytic leukemia, and Waldenström’s macroglobulinemia | no | Studies in xenograft models bearing human lymphoma cells showed activity of ibrutinib. (one model) (Pharmacology Reviews, Application Nr. 205552Orig1s000, FDA) |
| pomalidomide | thalidomide analogue indicated for the treatment of patients with multiple myeloma | no | SCID mice were given Raji BL cells via the tail vein to induce systemic lymphoma.  *In vivo* angiogenesis matrigel plug assay was used to show effect of pomalidomide on angiogenesis. (two models) (Pharmacology Reviews, Application Nr. 204026Orig1s000, FDA) |
| mechlorethamine | alkylating agent indicated for the topical treatment of Stage IA and IB mycosis fungoides‐type cutaneous T‐cell lymphoma | no | No animal models were reviewed, mechlorethamine has been used without FDA approval for nearly 50 years as a topical treatment to MF, a form of cutaneous T-cell lymphoma.  (Clinical Pharmacology and  Biopharmaceutics Reviews, Application Nr:  202317  Orig1s000, FDA) |

| 2014 | | | |
| --- | --- | --- | --- |
| ramucirumab | human vascular endothelial growth factor receptor 2 antagonist indicated for the treatment of advanced gastric cancer and non-small cell lung cancer | no | Matrigel plug assay *in vivo* was used to test blood vessel formation inhibition, and xenograft models to show anti-tumor activity have been used. (two models) (Pharmacology Reviews, Application Nr. 125477Orig1s000, FDA) |
| pembrolizumab | human programmed death receptor-1-blocking antibody indicated for the treatment of metastatic melanoma, metastatic non-small cell lung cancer, and recurrent or metastatic head and neck squamous cell carcinoma | Yes, first test designed to detect PD-L1 expression in non-small cell lung tumors (PD-L1 IHC 22C3 pharmDx). | The potential for PD-1 inhibition to suppress tumor growth was assessed in murine tumor models of colon adenocarcinoma using a murine surrogate molecule (antimouse-  PD-1 mAb). (one model) (Pharmacology Reviews, Application Nr. 125514Orig1s000, FDA) |
| olaparib | first-in-class oral poly ADP ribose polymerase inhibitor for the treatment of advanced ovarian cancer | Yes, BRAC Analysis CDx, that will detect the presence of mutations in the BRCA genes in blood samples. | Patient derived tumors were used in xenograft models to shown anti-tumor activity. (one model) (Pharmacology Reviews, Application Nr. 206162Orig1s000, FDA) |
| nivolumab | programmed death receptor-1 blocking antibody for the treatment of advanced melanoma | PD-L1 IHC 28-8 pharm DX test must be applied to test PD-L1 status. | Mice xenograft models were used successfully to show efficacy. (one model) (Pharmacology Reviews, Application Nr. 125554Orig1s000, FDA) |
| ceritinib | kinase inhibitor indicated for the treatment of patients with anaplastic lymphoma kinase-positive metastatic non-small cell lung cancer | Yes ALK test, but not developed simultaneous, as assays have already been available. | Certinib was effective in xenograft models. (one model) (Pharmacology Reviews, Application Nr. 205755Orig1s000, FDA) |
| belinostat | histone deacetylase inhibitor for the treatment of peripheral T-cell lymphoma | no | A xenograft model of ascites was used. (one model) (Pharmacology Reviews Application Nr:  206256Orig1s000, FDA) |
| blinatumomab | bispecific CD19-directed CD3 T-cell engager indicated for the treatment of Philadelphia chromosome-negative relapsed or refractory B-cell precursor acute lymphoblastic leukemia | Yes, testing for Philadelphia chromosome is required. | *In vivo* anti-tumor activity of blinatumomab was shown in several xenograft models in nude mice including human sarcoma cell lines. (one model)  (Pharmacology Reviews, Application Number:761038Orig1s000, FDA) |
| idelalisib | phosphoinositide 3-kinase delta inhibitor for the treatment of chronic lymphocytic leukemia, relapsed follicular B-cell non-Hodgkin lymphoma, and relapsed small lymphocytic lymphoma | no | No animal models were used, efficacy was only shown in different cell lines *in vitro.* (Pharmacology Reviews, Application Nr. 205858Orig1s000, FDA) |
| 2015 | | | |
| alectinib | oral anaplastic lymphoma kinase (ALK) inhibitor for the treatment of people with ALK-positive, locally advanced or metastatic non-small cell lung cancer (NSCLC) who have progressed on or are intolerant to crizotinib | Yes, ALK status must be tested, using an already available assay. | Xenograft models of NSCLC were used to show efficacy. (one model) (Pharmacology Reviews, Application Nr. 208434Orig1s000, FDA) |
| cobimetinib | kinase inhibitor indicated for use in combination with vemurafenib for the treatment of advanced melanoma with a BRAF V600E or V600K mutation | Yes, Health care providers should confirm the presence of BRAF V600 E or V600K mutation in their patients’ tumor specimens using one of the available FDA approved tests prior to starting treatment with cobimetinib in combination with vemurafenib. | Antitumor efficacy in BRAF and KRAS mutant human xenograft tumor models was shown. (one model) (Pharmacology Reviews, Application Nr. 206192Orig1s000, FDA) |
| palbociclib | cyclin-dependent kinase 4/6 inhibitor for the combination treatment of ER+, HER2- metastatic breast cancer | ER+, HER2- testing is needed. Tests were not newly developed, but FDA approved tests exist. | Xenograft models based on cell lines and patient derived tumors have been used to show anti-tumoral activity. (two models) (Pharmacology Reviews, Application Nr. 207103Orig1s000, FDA) |
| talimogene laherparevec | genetically modified oncolytic viral therapy indicated for the local treatment of melanoma lesions in the skin and lymph nodes | no | Xenograft models using cell lines and syngeneic mice models were used successfully to show efficacy.  (two models) (Pharmacology-Toxicology Review STN# 125518.000, FDA) |
| lenvatinib | oral multiple receptor tyrosine kinase inhibitor for the treatment of progressive radioiodine-refractory differentiated thyroid cancer and advanced renal cell carcinoma | no | Lenvatinib shows significant antitumor activity in human thyroid cancer xenograft models. (one model)  (Pharmacology Reviews, Application Nr. 206947Orig1s000, FDA) |
| trifluridine and tipiracil | thymidine phosphorylase inhibitor and nucleoside metabolic inhibitor combination indicated for the treatment of patients with metastatic colorectal cancer who are no longer responding to other therapies | no | Trifluridine and tipiracil exhibited  *in vivo* anti-tumor activity in various human colorectal cancer xenograft models in nude  mice. (one model)  (Pharmacology Reviews, Application Nr: 207981Orig1s000, FDA) |
| sonidegib | hedgehog pathway inhibitor indicated for the treatment of locally advanced basal cell carcinoma | no | The ability of  sonidegib to inhibit hair growth on C57BL/6 mice was examined, since hair follicles have active hedgehog signaling and inhibition of the smoothened receptor has been shown to interfere with the generation of hair follicles *in vitro.*  (one model)  (Pharmacology Reviews, Application Nr. 205266Orig1s000, FDA) |
| irinotecan liposome injection | liposomal formulation of the topoisomerase I inhibitor irinotecan indicated for the treatment of post-gemcitabine metastatic adenocarcinoma of the pancreas | no | Antitumor efficacy was tested in several xenograft models. (one model)  (Pharmacology Reviews, Application Nr: 207793Orig1s000, FDA) |
| necitumumab | epidermal growth factor receptor antagonist indicated in combination with gemcitabine and cisplatin for the treatment of patients with metastatic squamous non-small cell lung cancer | no | Combination with gemcitabine and cisplatin leads to significantly inhibition of tumor growth in nu/nu mice xenograft models. (one model) (Pharmacology Reviews, Application Nr. 125547Orig1s000, FDA) |
| osimertinib | tyrosine kinase inhibitor of epidermal growth factor receptor (EGFR) indicated for the treatment of patients with metastatic EGFR T790M mutation-positive non-small cell lung cancer whose disease has worsened on or after EGFR-TKI therapy | Yes, companion diagnostic cobas® EGFR Mutation Test v2 to detect EGFR mutations in patients with non-small cell lung cancer, including T790M needs to be applied. | Osimertinib exhibited greater anti-tumor activity in murine tumor models that are predominantly driven by mutant EGFR isoforms, including T790M, L858R, or exon 19 deletion mutants, than  in those that express wild-type EGFR, a  finding that correlated with the increased biochemical activity of osimertinib against  EGFR mutants relative to wild type EGFR. Further a xenograft model of brain metastasis was used.  (two models) (Pharmacology Reviews, Application Nr: 208065Orig1s000, FDA) |
| dinutuximab | chimeric monoclonal antibody used for the treatment of pediatric neuroblastoma | no | Dinutuximab suppressed the establishment as well as growth of progressively growing, established human neuroblastoma tumors in nude mice. (one model) (Pharmacology Reviews, Application Nr. 125516Orig1s000, FDA) |
| trabectedin | cytotoxic antitumor agent for the treatment of unresectable or metastatic liposarcoma or leiomyosarcoma, two common subtypes of soft tissue sarcoma | no | Xenograft  experiments show that trabectedin is cytotoxic to a variety of tumor cell lines, including cell lines derived from sarcomas. (one model) (Pharmacology Reviews, Application Nr. 207953Orig1s000, FDA) |
| daratumumab | human anti-CD38 monoclonal antibody indicated for the combination treatment of patients with multiple myeloma who have received at least one prior therapy | no | Daratumumab is efficacious in SCID mice xenograft models. (one model) (Pharmacology Reviews, Application Nr. 761036Orig1s000, FDA) |
| elotuzumab | Signaling Lymphocyte Activation Molecule-directed immunostimulatory antibody indicated in combination with lenalidomide and dexamethasone for the treatment of patients with multiple myeloma who have received one to three prior therapies | no | Xenograft mouse models were used to show efficacy. (one model) (Pharmacology Reviews, Application Nr. 761035Orig1s000, FDA) |
| panobinostat | histone deacetylase inhibitor for the combination treatment of multiple myeloma | no | Anticancer activity  of panobinostat was demonstrated in xenograft models of cancer (one model) (Pharmacology reviews, Application Nr: 205353Orig1s000, FDA) |
| ixazomib | oral proteasome inhibitor indicated for the use in combination with lenalidomide and dexamethasone for the treatment of patients with multiple myeloma who have received at least one prior therapy | no | Human xenograft mouse model  of multiple myeloma was used to show efficacy (one model) (Pharmacology Reviews, Application Nr. 208462Orig1s000, FDA) |
| 2016 | | | |
| cabozantinib | tyrosine kinase inhibitor indicated for the treatment of patients with advanced renal cell carcinoma who have received prior anti­-angiogenic therapy | no | Xenograft models were used to show antitumoral activity of cabozantinib (one model) (Pharmacology Reviews, Application Nr. 203756Orig1s000, FDA) |
| olaratumab | platelet-derived growth factor receptor alpha blocking antibody indicated in combination with doxorubicin for the treatment of patients with advanced soft tissue sarcoma | no | Data not available. (Pharmacology Reviews, Application Nr. 761038Orig1s000, FDA) |
| atezolizumab | programmed death-ligand 1 blocking antibody indicated for the treatment of patients with advanced urothelial carcinoma and patients with metastatic non-small cell lung cancer | Yes, FDA also approved the Ventana PD-L1 (SP142) assay to detect PD-L1 protein expression levels on patients’ tumor-infiltrating immune cells and help physicians determine which patients may benefit most from treatment with atezolizumab. | Anti-tumor activity of anti-PD-L1 antibody was shown in syngeneic mouse models using different cell lines. (one model)  (Pharmacology Reviews, Application Nr. 761034Orig1s000, FDA) |
| rucaparib | poly (ADP-ribose) polymerase inhibitor indicated for the treatment of advanced mutant BRCA ovarian cancer | Yes, specific gene mutation (deleterious BRCA)  FoundationFocus CDxBRCA test (Foundation Medicine Inc) needs to be applied, which is the first next-generation sequencing diagnostic test for this population of patients. | Subcutanous and orthotopic xenograft models were used to show efficacy, further patient derived tumors were used in mice.  (three models) (Pharmacology Reviews, Application Nr. 209115Orig1s000, FDA) |
| venetoclax | oral B-cell lymphoma-2 inhibitor for the treatment of patients with chronic lymphocytic leukemia with 17p deletion | Yes, venetoclax is indicated for use after confirmation of 17p deletion through the use of the FDA-approved companion diagnostic Vysis CLL FISH probe kit. | The ability of venetoclax to suppress tumor growth *in vivo* was evaluated in a broad spectrum of hematological tumor xenografts established in immune-compromised mice. (one model) (Pharmacology Reviews, Application Nr. 208573Orig1s000, FDA) |

Table S3: Approvals for psychiatric drugs 2012-2016

| Drug | Description | Companion diagnostic | Animal models for efficacy |
| --- | --- | --- | --- |
| 2012 | | | |
| 2013 | | | |
| vortioxetine | multimodal antidepressant for the treatment of major depressive disorder | no | Vortioxetine had an antidepressant-like effect in the forced swim test in mice, but not in the chronic mild stress model of depression. It had an anxiolytic-like effect in  the mouse marble burying test, the rat social interaction test, and in conditioned fear-induced vocalization model in rats (seven models, thereof three negative)  (Pharmacology Reviews, Application Nr: 204447Orig1s000, FDA) |
| levomilnacipran | serotonin and norepinephrine reuptake inhibitor indicated for the treatment of major depressive disorder | no | The anxiolytic-like effect of levomilnacipran was evaluated in male Sprague-Dawley  rats in two studies by testing the ability of the drug to attenuate conditioned stress-induced ultrasonic vocalizations.  The efficacy of levomilnacipran was also evaluated for its anxiolytic-like effects in  several other animal models such as Vogel Conflict test and Elevated Plus-Maze test  in male Han Wistar rats and Four Plates test  and Marble burying test  in male NMRI mice. The results of single-dose and chronic doses studies showed no anxiolytic-like effects by levomilnacipran in any of the animal models at any of the dose levels tested in the learned helplessness model of depression in male Wistar rats. Sub-  chronic administration of levomilnacipran failed to produce improvements in the escape performance in the active avoidance task demonstrating  the lack of antidepressant-like effects.  Forced swim test was positive (eleven models, thereof five negative)  (Pharmacology Reviews, Application Nr: 204168Orig1s000, FDA) |
| 2014 | | | |
| 2015 | | | |
| brexpiprazole | serotonin-dopamine activity modulator for the treatment of schizophrenia and the adjunctive treatment of major depressive disorder | no | Correlation was found in different animal models, effect was higher in combination therapy with fluoxetine,  paroxetine or sertraline (five models) (Pharmacology reviews, Application Nr. 205422Orig1s000,  205422Orig2s000, FDA |
| cariprazine | dopamine D3/D2 receptor partial agonist atypical antipsychotic for the acute treatment of manic or mixed episodes associated with bipolar I disorder and for the treatment of schizophrenia | no | Correlation with different animal models for antipsychotic like activity, antimanic potential and antidepressive like effects were reviewed. (17 models, thereof four negative)  (Pharmacology Reviews, Application Nr: 204370Orig1Orig2s000, FDA) |
| 2016 | | | |
| pimavanserin | non-dopaminergic, selective serotonin inverse agonist for the treatment of psychosis associated with Parkinson’s disease | no | Pimavanserin  pretreatment of rats  significantly reversed  the number of head twitches that were induced with 6-hydroxydopamine lesions, significantly reduced the amount of  amphetamine-induced  hyperactivity, and  significantly disrupted  prepulse inhibition  induced by the 6-hydroxydopamine  lesions (eight models) (Pharmacology Reviews, Application Nr: 207318Orig1s000, FDA |

Table S4: Approvals for anti-viral drugs 2012-2016

| Drug | Description | Companion diagnostic | Animal models for efficacy |
| --- | --- | --- | --- |
| 2012 | | | |
| elvitegravir, cobicistat, emtricitabine, tenofovir disoproxil fumarate | complete once-daily single tablet regimen for HIV-1 infection for treatment-naive adults | Yes, indicated for patients, who are antiretroviral treatment-naive  and have no known substitutions associated with resistance to the individual components of the regimen. | No animal models used for efficacy, this is not requested by the FDA. (Microbiology Reviews; Application Nr: 203100Orig1s000, FDA) |
| 2013 | | | |
| simeprevir | protease inhibitor for the treatment of chronic hepatitis C virus infection | Yes, screening of patients with HCV genotype 1a infection for the presence of virus with the NS3 Q80K polymorphism at baseline is strongly  recommended. Alternative therapy should be considered for patients  infected with HCV genotype 1a containing the Q80K polymorphism. | No animal models were reported, this is not requested by the FDA. (Microbiology Reviews; Application Nr: 205123Orig1s000, FDA) |
| sofosbuvir | once-daily oral nucleotide analogue for the treatment of chronic hepatitis C virus infection | Yes, therapy scheme depends on the genotype of virus and patient group. | No animal models were used, this is not requested by the FDA. (Microbiology Reviews; Application Nr: 204671 Orig1s000, FDA) |
| dolutegravir | integrase inhibitor indicated for use in combination with other antiretroviral agents for the treatment of HIV-1 | Yes, testing for different resistances:  Poor virologic response was observed in subjects  with an INSTI-resistance Q148 substitution plus 2 or  more additional INSTI-resistance substitutions including L74I/M,  E138A/D/K/T, G140A/S, Y143H/R, E157Q, G163E/K/Q/R/S, or  G193E/R. | No animal models used,this is not requested by the FDA. (Microbiology Reviews; Application Nr: 204790 Orig1s000, FDA) |
| 2014 | | | |
| ledipasvir and sofosbuvir | once-daily NS5A inhibitor and nucleotide analog polymerase inhibitor fixed-dose combination for the treatment of chronic hepatitis C virus genotype 1 | Yes, genotyping is needed. Drug is indicated for patients with chronic hepatitis C with genotype 1 | No animal models reported, this is not requested by the FDA. (Microbiology Reviews; Application Nr: 205834 Orig1s000, FDA |
| peramivir injection | influenza virus neuraminidase inhibitor indicated for the treatment of acute uncomplicated influenza in adults | Prescribers should consider  available information from the CDC on influenza  virus  drug susceptibility patterns and treatment  effects when deciding whether to use  RAPIVAB, but no obligatory testing. | Peramivir is active in mouse, ferret and cynomolgus monkey models of influenza  virus infections. (three models) (Microbiology/Virology reviews, Application Nr: 206426Orig1s000, FDA) |
| ombitasvir, paritaprevir, ritonavir and dasabuvir | NS5A inhibitor, NS3/4A protease inhibitor and CYP3A inhibitor combination co-packaged with a non-nucleoside NS5B palm polymerase inhibitor for the treatment of patients with genotype 1 chronic hepatitis C virus infection | Yes, genotyping is required, as the drug is indicated  for the treatment of  patients with genotype 1 chronic hepatitis C virus (HCV) infection including  those with compensated cirrhosis. | No animal studies were reported, this is not requested by the FDA. (Microbiology/Virology Reviews, Application Nr. 206619Orig1s000, FDA) |
| 2015 | | | |
| daclatasvir | NS5A inhibitor indicated for use in combination with sofosbuvir for the treatment of chronic hepatitis C virus genotype 1 or genotype 3 infection | Yes, genotype determination needed (drug is indicated for genotype 3). | No animal models were used, this is not requested by the FDA. (Microbiology/Virology Reviews, Application Nr. 206843Orig1s000, FDA) |
| elvitegravir, cobicistat, emtricitabine, and tenofovir alafenamide | antiretroviral combination for the treatment of HIV infection | Yes, resistance testing, indicated for patients twelve years of age and older who have no  antiretroviral treatment history or to replace the current antiretroviral  regimen in those who are virologically-suppressed (HIV-1 RNA less  than 50 copies per mL) on a stable antiretroviral regimen for at least six months with no history of treatment failure and no known substitutions  associated with resistance to the individual components of the drug. | No animal models used, this is not requested by the FDA. (Microbiology/Virology Reviews, Application Nr. 207561Orig1s000, FDA) |
| 2016 | | | |
| sofosbuvir and velpatasvir | nucleotide analog polymerase inhibitor and pan-genotypic NS5A inhibitor fixed-dose combination for the treatment of chronic genotype 1-6 hepatitis C virus infection | Yes, as treatment differs in patients 1) without cirrhosis and  patients with compensated cirrhosis  (Child  -Pugh A)  And 2) with decompensated  cirrhosis (Child  -Pugh B or C). | No animal models used, this is not requested by the FDA. (Microbiology/Virology Reviews, Application Nr. 208341 Orig1s000, FDA) |
| elbasvir and grazoprevir | once-daily, single tablet, NS5A replication complex inhibitor and NS3/4A protease inhibitor combination for the treatment of chronic hepatitis C virus genotype 1 and 4 infections | Yes, genotype must be determined. | Studies in chimpanzees have been performed, but were not requested. (one model) (Microbiology/Virology reviews, Application Nr: 208261  Orig1s000, FDA) |

Table S5: Approvals for anti-bacterial/fungal diseases 2012-2016

| Drug | Description | Companion diagnostic | Animal models for efficacy |
| --- | --- | --- | --- |
| 2012 | | | |
| bedaquilinie | oral diarylquinoline antimycobacterial drug indicated for the treatment of pulmonary multi-drug resistant tuberculosis | Yes, obtain susceptibility information for the background regimen against *Mycobacterium*  *tuberculosis* isolate if possible. | The bactericidal and sterilizing activity of bedaquiline as monotherapy and in  combination with first line drugs was investigated in the murine model. (one model) (Briefing package,  Division of Anti-Infective Products  Office of Antimicrobial Products  CDER, FDA,NDA: 204-384) |
| 2013 | | | |
| luliconazole | azole antifungal indicated for the topical treatment of interdigital tinea pedis, tinea cruris, and tinea corporis | Yes, testing for infections with *Trichophyton rubrum* and  *Epidermophyton*  *floccosum.* | Guinea pig models were successfully used to show efficacy. (three models) (Microbiology Reviews, Application Nr: 204153Orig1s000, FDA) |
| 2014 | | | |
| dalbavancin | second generation lipoglycopeptide antibiotic for the treatment of adult patients with complicated skin and skin structure infections, including those caused by methicillin-resistant *Staphylococcus aureus* | Yes, indicated for infections caused by susceptible isolates of the  following Gram-  positive microorganisms:  *Staphylococcus aureus*,  *Streptococcus pyogenes*,  *Streptococcus agalactiae*,  *Streptococcus dys*  *galactiae, Streptococcus anginosus*  group and  *Enterococcus faecalis* (vancomycin susceptible strains).  Two devices are approved by the FDA, but not mentioned in package insert. | Dalbavancin was efficacious in a number of animal infection studies, at lower and less frequent doses than comparators, and was in some cases efficacious with a single dose. Models included  acute septicemia in mice (a prophylaxis model) induced by intraperitoneal (IP) injection of *S. aureus, S. pyogenes* and other pathogens, and organ- or site -specific infections such as granuloma pouch (in rats), neutropenic mouse thigh, endocarditis (in rats and rabbits) and pneumonia (in rats). (six models) (Microbiology Reviews, Application Nr: 021883Orig1s000, FDA) |
| efinaconazole | topical triazole antifungal for the treatment of onychomycosis of the toenails | Yes, testing for the species *Trichophyton rubrum* and *Trichophyton mentagrophytes.* | Guinea pigs with tinea pedis were used successfully. (one model) (Microbiology Reviews, 203567Orig1s000, FDA) |
| tavaborole | oxaborole antifungal indicated for the topical treatment of onychomycosis of the toenails | Yes, testing for the species *Trichophyton rubrum* or *Trichophyton mentagrophytes.* | Only murine models of systemic infections were used and tavaborole failed to prevent death of the infected animals, but this was not relevant for the approval as onychomycosis should be treated, not systemic infection (not performed). (Microbiology/Virology Reviews, Application Nr: 204427Orig1s000, FDA) |
| oritavancin | semi-synthetic lipoglycopeptide antibiotic for the treatment of acute bacterial skin and skin structure infections | Yes, indicated for the treatment of adult patients with acute bacterial skin and skin structure infections caused or suspected to be caused by susceptible  isolates of the following Gram  -positive microorganisms:  *Staphylococcus aureus*,  *Streptococcus*  *pyogenes,*  *Streptococcus agalactiae,*  *Streptococcus* *dysgalactiae,*  *Streptococcus*  *anginosus*  group  and  *Enterococcus faecalis*  (vancomycin  -susceptible isolates only). | The efficacy of oritavancin has been investigated in a number of animal models of infection including  1) staphylococci and enterococci bloodstream infections in mice; 2) endocarditis models of staphylococci and enterococci infections in rabbits and rats; 3) mouse and rat  *S. pneumoniae*  infection models; 4) biofilm *S. aureus*  infection models in  mouse; 5) meningitis models of *S. pneumonia*  infection in rabbits; and 6) *B. anthracis*  mouse infection models. (six models) (Microbiology/Virology Reviews, Application Nr: 206334Orig1s000, FDA) |
| tedizolid phosphate | oxazolidinone antibiotic drug indicated for the treatment of acute bacterial skin and skin structure infections | Yes, susceptibility testing is strongly recommended, active against *Staphylococcus aureus*, *Streptococcus pyogenes*, *Streptococcus agalactiae, Streptococcus anginosus* Group, and *Enterococcus faecalis.* | The applicant has provided data from a variety of animal models including 1) staphylococcal systemic  infections in mice; 2) enterococcal systemic infections in mice; 3)  streptococcal systemic infections in mice; 4)  MRSA skin and soft tissue infection in mice; 5) mouse thigh infection model with MRSA and MSSA; 6) rat skin and soft tissue infection; 7) lung infection and epithelial lining fluid exposure in mice; 8) a neutropenic  mouse pneumonia model; 9) an *S. aureus*  endocarditis model in rabbits; 10) and a mouse *Streptococcus*  *pneumoniae* model.  Efficacy was demonstrated in all models tested. (ten models) (Micrology/Virology Reviews, Application Nr. 205436Orig1s000, FDA) |
| finafloxacin otic suspension | fluoroquinolone antimicrobial for the treatment of acute otitis externa, commonly known as swimmer’s ear | Yes, susceptibility testing should be performed, active against susceptible strains of  *Pseudomonas*  *aeruginosa*  and  *Staphylococcus aureus.* | Efficacy was tested in several models including acute otitis externa, sepsis models, respiratory tract infection, gastrointestinal and intra-abdominal infections, skin and soft tissue infections, and urinary tract infections using different animals and pathogens. (16 models) (Microbiology Reviews, 206307Orig1s000, FDA) |
| ceftolozane and tazobactam | cephalosporin and beta-lactamase inhibitor combination for the treatment of complicated intra-abdominal infections and complicated urinary tract infections | Yes, susceptibility should be tested. | Efficacy in different animal models was shown, including sepsis, pneumonia, urinary tract infection, burn wound infection, thigh infection in different animals and sing different pathogens. (13 models) (Microbiology/Virology Reviews, Application NR. 206829Orig1s000, FDA) |
| 2015 | | | |
| ceftazidime-avibactam | next generation, non-β lactam β-lactamase inhibitor and third-generation, antipseudomonal cephalosporin antibiotic combination for the treatment of complicated intra-abdominal infections and complicated urinary tract infections | Yes, should be used to treat only indicated infections that are proven or strongly  suspected to be caused by susceptible bacteria. | Efficacy was tested in several animal models, including murine systemic infection, pneumonia immune-compromised mice, pyelonephritis immune-compromised mice, meningitis immune-competent rabbit, and murine thigh infection. (five models) (Microbiology/ Virology Reviews, Application Nr: 206494Orig1s000, FDA) |
| isavuconazonium sulfate | azole antifungal indicated for the treatment of invasive aspergillosis and invasive mucormycosis | Yes, specimens for fungal culture and other relevant laboratory studies (including histopathology) to isolate and identify causative organism(s) should be obtained prior to initiating antifungal therapy. | Tested in different animal models including aspergillosis model, neutropenic murine model, non-neutropenic murine model, pulmonary aspergillosis model, and mucormycosis model in different animals using different pathogens . (eight models) (Microbiology/Virology Reviews, Application Nr: 207500Orig1s000 / 207501Orig1s000, FDA) |

Table S6: Approvals for monogenetic orphans 2012-2016

| Drug | Description | Companion diagnostic | Animal models for efficacy |
| --- | --- | --- | --- |
| 2012 | | | |
| ivacaftor | for the treatment of cystic fibrosis in patients age 6 years and older who have a G551D mutation in the CFTR gene | Yes, only indicated for patients with mutation in the CFTR gene. | Data not available. (Pharmacology Reviews, Application Nr: 203188Orig1s000, FDA) |
| Taliglucerase alfa | used as long-term enzyme replacement therapy in patients with Type 1 Gaucher disease | Yes, indicated for adults with a confirmed diagnosis of Type 1  Gaucher disease. | No animal models reviewed for efficacy. (Pharmacology Reviews, Application Nr: 022458Orig1s000, FDA) |
| 2013 | | | |
| factor XIII concentrate (human) | peri-operative management of surgical bleeding in adult and pediatric patients with congenital Factor XIII deficiency | Yes, dose adjustment using the Berichrom activity assay must be performed. | FXIII knockout mouse models were used successfully. (two models) (Summary of Non-clinical Studies in STN 125385/0, FDA) |
| coagulation factor IX (recombinant) | for routine prophylaxis to prevent or reduce the frequency of bleeding episodes in adults with Hemophilia B | Yes, titration of the dose based on the patient's clinical response and  individual pharmacokinetics, in particular incremental recovery and half-life is required. | Data not available. |
| 2014 | | | |
| elosulfase alfa | treatment of patients with Mucopolysaccharidosis type IVA (Morquio A syndrome) | Yes, determination of mucopolysaccharidosis type IVA (MPS IVA; Morquio A syndrome) | No animal models reported for efficacy. (Pharmacology Reviews, Application Nr. 125460Orig1s000, FDA) |
| metreleptin | adjunct to diet as replacement therapy to treat the complications of leptin deficiency in patients with congenital or acquired generalized lipodystrophy | no | Animal studies using lipodystrophic mice have been used to show the efficacy of leptin, metreleptin was not used. (one model) (Pharmacology Reviews, Application Nr: 125390Orig1s000, FDA) |
| coagulation factor IX (recombinant), Fc fusion protein | for control and prevention of bleeding episodes, perioperative management, and routine prophylaxis to prevent or reduce the frequency of bleeding episodes in adults and children with Hemophilia B | Yes, monitoring of the plasma Factor IX activity by performing the one  -stage clotting assay is required | Efficacy was shown in different mouse models. (four models) (Pharmacology -Toxicology Primary Discipline Review, July 28, 2013 – Alprolix, FDA) |
| antihemophilic factor (recombinant), Fc fusion protein) | treatment of adults and children with Hemophilia A (congenital Factor VIII deficiency) for control and prevention of bleeding episodes, perioperative management, and routine prophylaxis to prevent or reduce the frequency of bleeding episodes | Yes, performing of a validated test (e.g., one stage clotting  assay), to confirm that adequate Factor VIII levels have been achieved and maintained is required. | Genetically modified Factor VIII-deficient hemophilic mice and dogs, monkeys and rats were used successfully. (four models) (Final BLA Review Memorandum, File BLA 125487/0/0  (cross reference: IND 14134), FDA) |
| C1-esterase inhibitor (recombinant) | Treatment of acute attacks of hereditary angioedema in adult and adolescent patients | Yes, test for hereditary angioedema should be performed | No animal models for efficacy were reviewed in the FDA reviews. |
| 2015 | | | |
| human factor X | treatment of patients with hereditary Factor X Deficiency | Yes, measurement of post-infusion plasma Factor X levels for each patient before and after surgery is required, to ensure that hemostatic levels are obtained and maintained. | Nonclinical *in vivo* studies of primary pharmacodynamics were not performed in animals due to the lack of an available animal model for Factor X deficiency. (Pharmacology/Toxicology review File BLA 125506/0/0, FDA) |
| asfotase alfa | for the treatment of patients with perinatal/infantile-and juvenile-onset hypophosphatasia | Yes, testing of alkaline phosphatase activity for diagnosis is required. | Asfotase alfa prevents craniosynostosis (the premature fusion of cranial bones) and additional craniofacial skeletal abnormalities in Alpl(-/-) mice. (one model) (Summary Review, Application Nr. 125513Orig1s000, FDA) |
| recombinant von Willebrand factor (rhVWF) | indicated for on-demand treatment and control of bleeding episodes in adults diagnosed with von Willebrand disease | Yes, monitoring of plasma levels of VWF:RCo and factor VIII activities is required. | Nonclinical studies to assess the primary pharmacologic activity in hemostasis were conducted *in vivo* using congenitally VWF-deficient dogs, and mice that were genetically engineered to delete expression of the murine VWF gene (i.e., VWF-knockout) mice. (two models) (Pharmacology/Toxicology Secondary Review, STN BLA #: 125577/0, FDA) |
| sebelipase alfa | indicated for the treatment of patients with a diagnosis of lysosomal acid lipase deficiency | Yes, test for biallelic pathogenic variants in LIPA or deficient LAL enzyme activity in peripheral blood leukocytes, fibroblasts, or dried blood spots is required. | A rat model of lysosomal acid lipase deficiency was successfully used to show efficacy. (one model) (Pharmacology Reviews, Application Nr. 125561Orig1s000, FDA) |
| 2016 | | | |
| recombinant fusion protein linking coagulation factor IX with albumin (rIX-FP) | indicated for the on-demand control and prevention of bleeding episodes, perioperative management of bleeding, and routine prophylaxis to prevent or reduce the frequency of bleeding episodes | Monitoring of  Factor  IX plasma levels by a one-stage clotting assay is required to confirm that adequate FactorIX levels have been achieved and maintained. | A canine model of Hemophilia B  (i.e. dogs with a naturally occurring mutation and/or deletion of FIX function) and FIX knock-out mice have been used. (two models) (Pharmacology/Toxicology Primary Discipline Review, Original BLA STN 125582/0, FDA) |
| eteplirsen | treatment of Duchenne muscular dystrophy (DMD) in patients who have a confirmed mutation of the DMD gene that is amenable to exon 51 skipping | Yes, testing for patients who have a confirmed mutation of the DMD gene that is amenable to exon 51 skipping. | Studies in mdx mice and  CXMD dogs showed efficacy. (two models) (Pharmacology reviews, Application Nr: 206488Orig1s000, FDA) |
| lumacaftor/ivacaftor | treatment of cystic fibrosis in patients age 6-11 year old who are homozygous for the F508del mutation in the CFTR gene | Yes, test for patients which are homozygous for the F508del mutation in the CFTR gene. | Ivacaftor was approved before, for lumacaftor no animal models were reported in the reviews. (Summary Review, Application Nr: 206038Orig1s000, FDA) |
| nusinersen | treatment of spinal muscular atrophy in pediatric and adult patients | Conduct the following laboratory tests at baseline and prior to each dose of nusinersen and as clinically needed:  Platelet count  Prothrombin time, activated partial thromboplastin time,  Quantitative spot urine protein testing | In spinal muscular atrophy mouse models nusinersen appeared to promote SMN2 exon 7 splicing, showing activity at tissue concentrations achieved  clinically. (one model) (Pharmacology Reviews, Application Nr: 209531Orig1s000) |
